# *Lactobacillus rhamnosus* GR-1 Alleviates *Escherichia coli*-Induced Inflammation *via* NF-κB and MAPKs Signaling in Bovine Endometrial Epithelial Cells

**DOI:** 10.3389/fcimb.2022.809674

**Published:** 2022-03-02

**Authors:** Jiawei Liu, Xiaowei Feng, Botong Li, Yan Sun, Tianxiong Jin, Mingque Feng, Yaodi Ni, Mingchao Liu

**Affiliations:** College of Veterinary Medicine, Hebei Agricultural University, Baoding, China

**Keywords:** bovine endometrial epithelial cell, inflammatory response, *Lactobacillus rhamnosus*, *Escherichia coli*, NF-κB, MAPKs

## Abstract

*Escherichia coli* counts as a major endometritis-causing pathogen among dairy cows, which lowered the economic benefits of dairy farming seriously. Probiotic consumption has been reported to impart beneficial effects on immunomodulation. However, the inflammatory regulation mechanism of probiotics on endometritis in dairy cows remains unexplored. The current work aimed to clarify the mechanism whereby *Lactobacillus rhamnosus* GR-1 (*L. rhamnosus* GR-1) resists bovine endometrial epithelial cells (BEECs) inflammatory injury induced by *E. coli*. The model of cellular inflammatory injury was established in the BEECs, which comes from the uterus of healthy dairy cows using *E. coli*. The outcome of *L. rhamnosus* GR-1 addition on inflammation was evaluated in BEECs with *E. coli*-induced endometritis. The underlying mechanisms of anti-inflammation by *L. rhamnosus* GR-1 were further explored in *E. coli*-stimulated BEECs. In accordance with the obtained results, the use *L. rhamnosus* GR-1 alone could not cause the change of inflammatory factors, while *L. rhamnosus* GR-1 could significantly alleviate the expression of *E. coli*-induced inflammatory factors. Based on further study, *L. rhamnosus* GR-1 significantly hindered the TLR4 and MyD88 expression stimulated by *E. coli*. Moreover, we observed that in BEECs, *L. rhamnosus* GR-1 could inhibit the *E. coli*-elicited expressions of pathway proteins that are associated with NF-κB and MAPKs. Briefly, *L. rhamnosus* GR-1 can effectively protect against *E. coli*-induced inflammatory response that may be closely related to the inhibition of TLR4 and MyD88 stimulating NF-κB and MAPKs.

## Introduction

Endometritis refers to the inflammation of the uterus, which is more likely to occur in a fresh cow. This disease exerts a certain impact on the pregnancy percentage and milk output of dairy cows (Sheldon et al., 2006), and it significantly lowers the economic benefits derived from dairy cow breeding. Generally, endometritis in dairy cows is caused by various bacteria in their uterine cavity at the early stage of postpartum. *Escherichia coli* is the main pathogen that causes endometritis in dairy cows. Currently, with the widespread application of antibiotic therapy, problems such as drug resistance are becoming increasingly serious. Therefore, it is essencial to formulate a novel treatment strategy with high efficiency and no side effects (Yah and Simate, 2015). A large body of literature has proved that lactic acid bacteria (LAB) can prevent inflammation in the gastrointestinal tract and genitourinary tract (Reid et al., 2001; Collado et al., 2009). Synthesis of lactic acid, organic enzymes, hydrogen peroxide, acetic acid, and other substances from the metabolic reaction of LAB (Blajman et al., 2015; Huang et al., 2015; Sgibnev and Kremleval, 2015) can enhance the integrity of epithelial cells, improve the activity of immune cells, and competitively destroy pathogenic microorganisms, suggesting that LAB can prevent and treat endometritis (Coudeyras et al., 2008; Parolin et al., 2015). Most importantly, the pronounced effect of LAB in the treatment and prevention of endometritis in dairy cows has been demonstrated (Deng et al., 2015; Genís et al., 2016). At present, LAB has been isolated from the intestinal and reproductive tracts of humans or livestock and poultry (Turner et al., 2014; Li et al., 2021; Shams et al., 2021). For example, *Lactobacillus rhamnosus* GR-1, which is urethral isolate from normal females (Chan et al., 1984), has been applied to prevent bacterial vaginosis and premature delivery. The above applications emphasize that *L. rhamnosus* GR-1 can modulate immunity and resist microbes (Reid, 2001; Kim et al., 2005; Yang et al., 2014). However, the exact mechanisms behind the impact of *L. rhamnosus* GR-1 on the inflammatory response remain unclear.

Specific identification of type-I transmembrane proteins for pathogen-associated molecular patterns is possible in terms of Toll-like receptors (TLRs) (Chen et al., 2016). There are several types of TLRs, of which TLR4 acts as a cell-surface model receptor primarily responsible for identifying lipopolysaccharides. It is the key immune receptor molecule that activates the immune system (Samuelsson et al., 2004). Eventually, such recognition could elicit the initiation of the MyD88 pathway and then the downstream MAPKs and NF-κB, which is followed by release of diverse inflammation-associated cytokines such as TNF-α and IL-1β. Thus, involvement of these signaling pathways in the inflammatory system is possible (Maisonneuve et al., 2014; Zhang et al., 2017; Qiao et al., 2020). According to the related studies, some LAB can achieve immunity modulation and inflammation mitigation *via* pathways like NF-κB and MAPKs (Zhou et al., 2018; Mao et al., 2020; Yang et al., 2021).

Although several scholars have verified that a few *Lactobacillus* strains are encouragingly effective in regulating bodily health, the anti-inflammation regulation mechanism of LAB on bovine endometrial epithelial cells (BEECs) remains to be explored to a large extent. In this study, *L. rhamnosus* GR-1 was used to treat the BEECs induced by *E. coli*. Meanwhile, the mRNA expressions of TNF-α, IL-6 IL-8 and IL-1β, and also the expression of the key proteins of TLR4, MyD88, MAPK and NF-κB pathways, were determined. The current work attempted to investigate the inflammation-associated impact of *L. rhamnosus* GR-1 on BEECs induced by *E. coli*.

## Materials and Methods

### Bacterial Strains

*L. rhamnosus* GR-1 strain (ATCC55826) was obtained from the Laboratory of Clinical Nutrition and Immunology, College of Veterinary Medicine, China Agricultural University. Cultivation of *L. rhamnosus* GR-1 was accomplished using the MRS (deMan Rogosa Sharp) medium (AOBOX, Beijing, China) under anaerobic and 37°C conditions for 24 h. Finally, through stepwise dilution, the culture concentration of 5 × 10^6^ CFU/ml was clarified, and the CFU (colony-forming unit) was calculated on the DEME/F12 basic medium (Gibco, Beijing, China).

The *E. coli* (O111: K58 [B4]) strains (CVCC1450) were obtained from the Laboratory of Clinical Nutrition and Immunology, College of Veterinary Medicine, China Agricultural University. *E coli* was inoculated in the LB broth medium (AOBOX, Beijing, China) and cultured at 180 rpm for 12 h in an oscillating incubator at 37°C. Subsequently, the regulation of the final bacterial density was assigned as 5 × 10^5^ CFU/ml.

### Cell Culture

Separation and identification of primary BEECs were accomplished in line with the prior procedures (Wang et al., 2020). The bovine uterus was obtained from healthy non-pregnant Holstein cows, which were slaughtered on a commercially-operated cattle farm. These uteri were immediately delivered to the laboratory after being placed in ice-cold sterilized PBS (phosphate-buffered saline). Following separation of purified primary BEECs, they were preserved in the 10% FBS (GEMINI, Shanghai, China) DMEM/F12 medium (Gibco, Beijing, China) involving penicillin/streptomycin (1%; Solarbio, Beijing, China). Cultivation of the cells was accomplished based on 25-cm^2^ flasks in a 37°C incubator with 5% CO_2_. Trypsin-EDTA (0.25%; Gibco, Beijing, China) was adopted for digesting the cultured cells, aiming to allow subsequent subcultivation or protein extraction.

### Cell Treatment

The experiment was assigned to four groups and subjected to different treatments: Group 1: normal control; Group 2: cultured BEECs treated with 5 × 10^5^ CFU/ml *E. coli*; Group 3: cultured BEECs treated with 5 × 10^6^ CFU/ml *L. rhamnosus* GR-1; Group 4: cultured BEECs pre-treated with 5 × 10^6^ CFU/ml *L. rhamnosus* GR-1 for 3 h and subsequently raised with 5 × 10^5^ CFU/ml *E. coli* for 6 h.

### Cell Viability Assay

Cell viability was evaluated with the application of the methylthiazol tetrazolium (MTT) (Solarbio, BeiJing, China) assay by inoculation of 1 × 10^5^ cells per ml in a 96-well plate (CELLTER, Santiago, Chile), and incubation under 5% CO_2_ and 37°C conditions. Afterwards, pretreatment of the cells proceeded using specific concentrations of *E. coli*. According to the time sequence of 12, 24, 36, 48, 60, and 72 h, the abandonment medium of the cell culture plate was removed at a different time point. Meanwhile, every culture well was added with the MTT solution. Thereafter, a 4-h incubation was accomplished for every mixture. Then, formazan (110 µl) was added at different time points, and the plate was slowly shaken for 10 min. Final step was the determination of the 490-nm cellular absorbance.

### Quantitative Real-Time Reverse Transcription Polymerase Chain Reaction (qRT-PCR)

Followed by *E. coli* infection, *L. rhamnosus* GR-1 was used to pretreat the cells. All treated cells were harvested at 6 h after infection, from which the total RNA was extracted based on Triquick Reagent (Solarbio, Beijing, China) for quantitative analysis *via* the Nanodrop instrument (Thermo Fisher Scientific, UK). Aided by FastKing-RT SuperMix (Tiangen, Beijing, China), synthesis of cDNA was accomplished from 1 μg of the acquired total RNA. In terms of quantitative real-time PCR, the SuperReal PreMix Plus (Tiangen, Beijing, China) and the subsequent LightCycler 480 system (Roche, Switzerland) were employed. As for the amplification specificity assessment, single-band recognition was performed at the anticipated molecular weight in the DNA agarose gel. Meanwhile, a unimodal peak was identified in the qRT-PCR melting plots. **Table 1** lists the primer sequences. Comparative threshold cycle approach was adopted for the computation of the data. Later, the IL-6, IL-8, IL-1β, MyD88, TNF-α, and TLR4 levels were examined.

**Table 1:** Target gene primer sequence, amplified fragment length, and sequence number.

Target gene		Primer sequence (5’-3’)	Product length (bp)	Serial number
IL-8	F	ACACATTCCACACCTTTCCAC	149	AF232704
	R	ACCTTCTGCACCGACTTTTC		
IL-6	F	GCTGAATCTTCCAAAAATGGAGG	215	NM_173923.2
	R	GCTTCAGGATCTGGATCAGTG		
IL-1β	F	CCTCGGTTCCATGGGAGATG	119	NM_174093.1
	R	AGGCACTGTTCCTCAGCTTC		
MyD88	F	AAGTACAAGCCAATGAAGAAAGAG	102	NM_001014382.2
	R	GAGGCGAGTCCAGAACCAG		
TNF-α	F	TCCAGAAGTTGCTTGTGCCT	144	NM_173966.3
	R	CAGAGGGCTGTTGATGGAGG		
TLR4	F	TATGAACCACTCCACTCGCTC	207	DQ839566
	R	CATCATTTGCTCAGCTCCCAC		
GAPDH	F	GTCTTCACTACCATGGAGAAGG	201	NM_001034034
	R	TCATGGATGACCTTGGCCAG		

### Western Blotting

After growth and challenge utilizing a 6-well format (CELLTER, Santiago, Chile), lysis of the cells was accomplished with the RIPA buffer (Solarbio, Beijing, China), followed by electrophoretic shift of the protein samples onto the PVDF (polyvinylidene difluoride) membranes (Solarbio, Beijing, China). The next step was a 1-h blockage of unbound PVDF sites with buffer and subsequent incubation based on optimal primary antibody dilutions, such as anti-phospho-NF-κB p65 & anti-NF-κB p65 (both Bioss, Beijing, China), anti-phospho-IκB alpha (Bioss, Beijing, China), anti-phospho-p38 MAPK & anti-phospho-ERK1/2 MAPK (both Cell Signaling Technology, MA, USA), anti-beta-Actin (Bioss, Beijing, China), anti-phospho-NF-κB p65 (Bioss, Beijing, China), anti-phospho-JNK1 (Bioss, Beijing, China), and anti-GAPDH (Proteintech, Wuhan, China) antibodies, for the proteins of interests for 12 h at 4°C. After rinsing four times with PBS-containing Tween 20 (PBST), a further 1-h incubation of the membranes proceeded with alkaline phosphatase (AP)-labeled secondary antibodies at 37°C. Then, the membranes were rinsed four times in PBST (Solarbio, BeiJing, China). A BCIP/NBT Substrate color kit was used for visualization.

### Statistical Analysis

Data represented are means ± SEMs (standard errors of means). One-way ANOVA (analysis of variance) in the IBM SPSS Statistics 21 was employed to make statistical comparisons. Differences are considered to present statistical significance at a threshold of *P <*0.05 or *P <*0.01. Graphs were plotted scientifically with the aid of GraphPad Prism 8. In addition, experiments were all triplicated.

## Results

### Viability of BEECs

**Figure 1:**
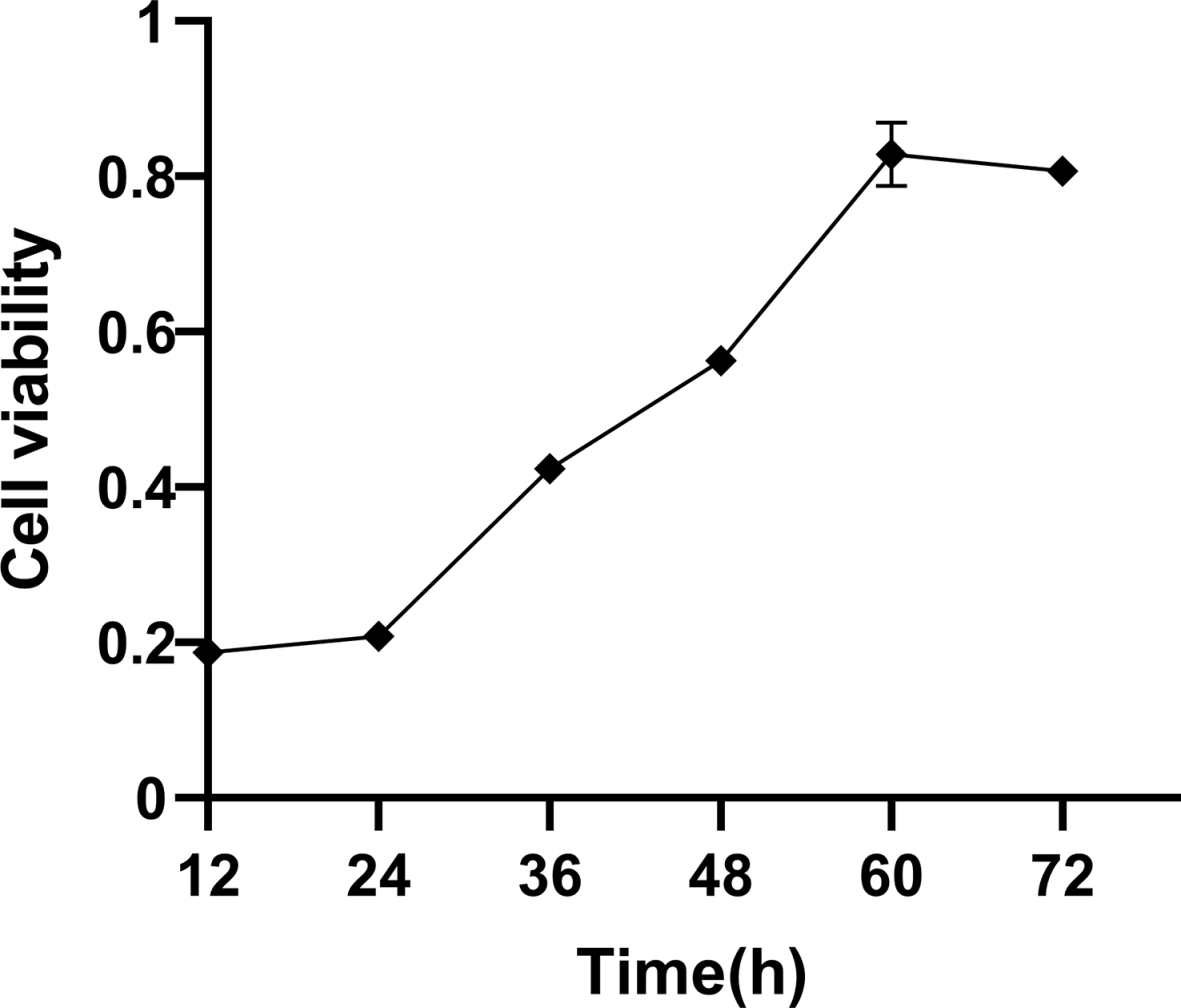
Cell viability in BEECs. Cell viability was detected by MTT at different periods. Data are presented as the mean ± SEM of three independent experiments.

In the current work, we examined how the temporal duration impacted the cellular viability. The results revealed that the growth rate of BEECs was slow during 12–24 h. During the period of 24 and 60 h, the growth rate of BEECs was rapid. At 60 and 72 h, the growth rate of BEECs was relatively slow (**Figure 1**). This observation was in consistence with the proliferation characteristics of BEECs.

### *L. rhamnosus* GR-1 Inhibits Pro-Inflammatory Gene Expression in *E. coli-*Stimulated BEECs

**Figure 2:**
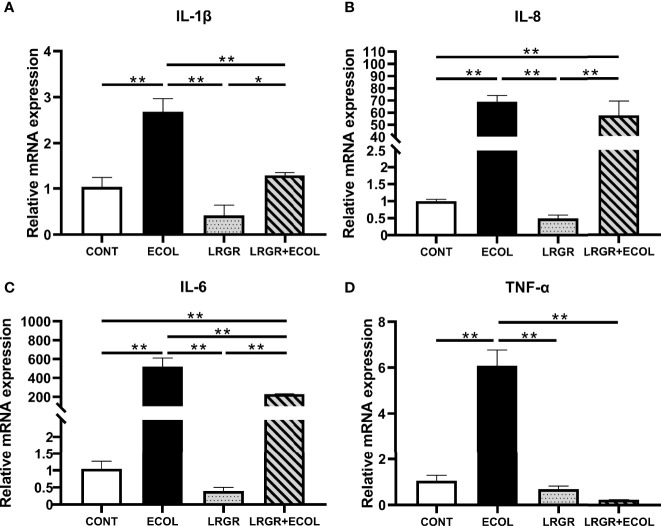
Effect of *L. rhamnosus* GR-1 on the mRNA expression of inflammatory factors in BEECs induced by *E*. *coli*. IL-1β, IL-8, IL-6, and TNF-α mRNA were quantified using the qPCR assay. **(A)** The expression of IL-1β mRNA, **(B)** IL-8 mRNA, **(C)** IL-6 mRNA, and **(D)** TNF-α mRNA. Data represented are means ± SEMs from triplicate experiments. *P <0.05, **P <0.01.

In terms of the inflammatory status assessment of BEECs, the proinflammatory cytokine levels were determined. Therefore, we evaluated whether *L. rhamnosus* GR-1 could inhibit the expression of pro-inflammatory genes containing IL-1β (**Figure 2A**), IL-8 (**Figure 2B**), IL-6 (**Figure 2C**), and TNF-α (**Figure 2D**) in BEECs.

The results revealed that these pro-inflammatory genes were upregulated by *E. coli* treatment (*P <*0.01). Difference was insignificant for the group treated using *L. rhamnosus* GR-1 alone (*P >*0.05). Furthermore, *L. rhamnosus* GR-1 pre-treatment could significantly inhibit the increased expression of these genes in *E. coli-*stimulated BEECs (*P <*0.01).

### *L. rhamnosus* GR-1 Inhibits TLR4 and MyD88 mRNA Expression in *E. coli-*Stimulated BEECs

**Figure 3:**
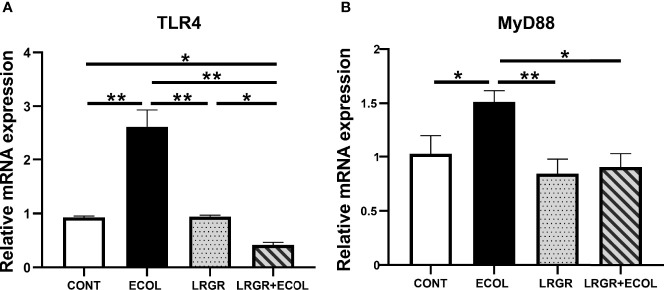
*L. rhamnosus* GR-1 affects the TLR4 and MyD88 expression in *E*. *coli*-induced BEECs. **(A)** Expression of TLR4 mRNA and **(B)** MyD88 mRNA. Data are presented as the mean ± SEM of three independent experiments. *P <0.05, **P <0.01.

To clarify the probable orchestrating pathways responsible for *E. coli*-elicited inflammation and *L. rhamnosus* GR-1-suppression inflammation in the BEECs, we concentrated on TLRs, which led to rapid inherent immunity provocation by triggering the proinflammatory cytokine releases. In particular, an LPS membrane receptor TLR4 exhibits rich expression in varying epithelial cells. As shown in **Figure 3**, in contrast to the controls, prominent upregulations of the TLR4 and MyD88 mRNA were found in the *E. coli*-elicited cells (*P* < 0.01, and *P* < 0.05, respectively). The difference was insignificant for the sole *L. rhamnosus* GR-1 treatment group (*P* > 0.05). However, based on **Figure 3**, the *L. rhamnosus* GR-1 treatment of cells led to drastically inhibited expressions of TLR4 and MyD88 (*P* < 0.01, and *P* < 0.05, respectively). Agreement of the obtained results was found with the inhibitory activity of *L. rhamnosus* GR-1 against TLR4 and MyD88 production described below.

### Impact of *L. rhamnosus* GR-1 on *E. coli*-Elicited BEECs Nuclear Factor κappa-B (NF-κB) Signaling Pathway Activity

**Figure 4:**
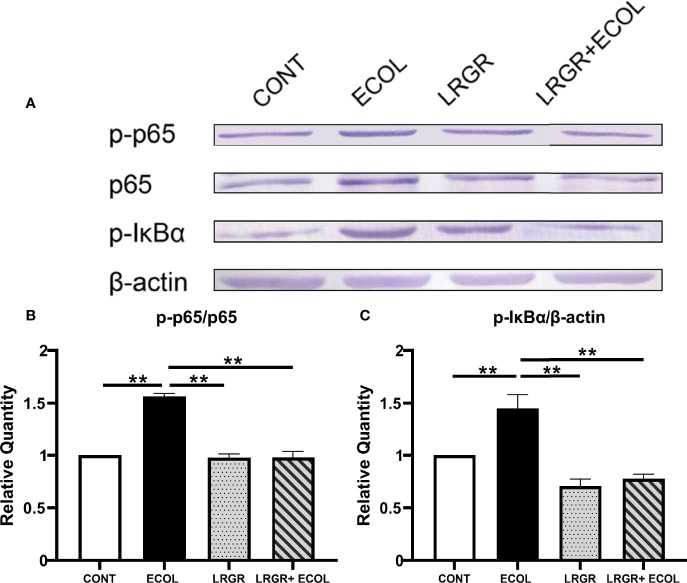
*L. rhamnosus* GR-1 affects the NF-κB pathway protein expression in *E*. *coli*-induced BEECs. The p-p65 and p-IκBα expressions in the *L. rhamnosus* GR-1- and *E*. *coli*-elicited BEECs were explored by Western blotting. **(A)** p-p65, p-IκBα. The resultant phosphorylated protein as percentages of total protein is averaged and plotted, **(B)** p-p65/p65 and **(C)** p-IκBα*/*β-actin. Data represented are means ± SEMs from triplicate experiments. **P <0.01.

Positive association of a few proinflammatory cytokine expressions was found with the NF-κB and MAPKs signalling initiation, since these cytokines were capable of eliciting the distal NF-κB and MAPKs pathways straightforwardly. Thus, to further investigate the anti-inflammatory action of *L. rhamnosus* GR-1 against inflammation elicited by *E. coli*, we examined the levels of NF-κB axis-associated proteins in BEECs challenged with *L. rhamnosus* GR-1 *E. coli*. As presented in **Figure 4**, compared with the control, treatment with *E. coli* in BEECs cells significantly enhanced the p-p65 and p-IκBα protein expressions (*P <*0.01). The difference was insignificant for the group treated using *L. rhamnosus* GR-1 alone (*P >*0.05). Furthermore, *L. rhamnosus* GR-1 pre-treatment could significantly inhibit the increased expression of the phosphorylation levels of p65 and IκBα in *E. coli-*stimulated BEECs (*P <*0.01).

### Impact of *L. rhamnosus* GR-1 on *E. coli*-Elicited BEECs MAPKs Signaling Pathway Activity

**Figure 5:**
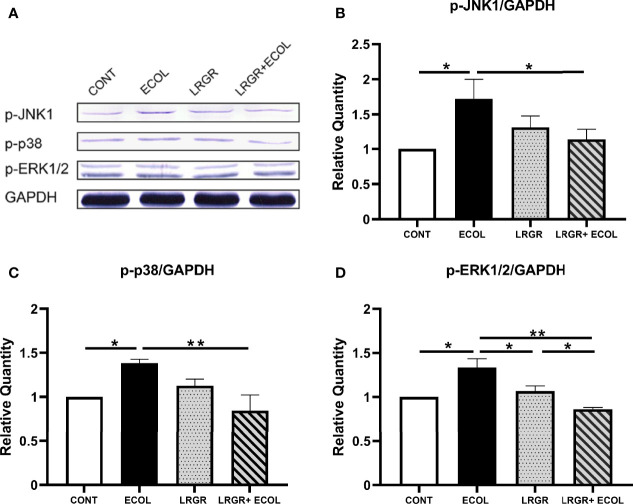
*L. rhamnosus* GR-1 affects the p-JNK1, p-p38, and p-ERK1/2 protein expression in *E*. *coli*-induced BEECs. The protein levels of p-JNK1, p-p38, and p-ERK1/2 in BEECs stimulated with *E*. *coli* and *L. rhamnosus* GR-1 was investigated by Western blotting. **(A)** p-JNK1, p-p38, p-ERK1/2. The resultant mean ratio of phosphorylated protein to total protein is depicted, **(B)** p-JNK1/GAPDH, **(C)** p-38*/*GAPDH and **(D)** p-ERK1/2/GAPDH. Data are presented as the mean ± SEM of three independent experiments. *P <0.05, **P <0.01.

Then, the related protein levels in the MAPKs axis were assessed in *L. rhamnosus* GR-1 and *E. coli*-induced BEECs. As presented in **Figure 5**, in contrast to the controls, treatment with *E. coli* in BEECs could significantly enhance the protein levels of p-JNK1, p-p38, and p-ERK1/2 (*P* < 0.01 to *P* < 0.05). The difference was insignificant for the group treated using *L. rhamnosus* GR-1 alone (*P >*0.05). Furthermore, *L. rhamnosus* GR-1 pre-treatment significantly repressed the phosphorylation level elevations of p-JNK1, p-p38, and p-ERK1/2 in *E. coli-*stimulated BEECs (*P* < 0.05, *P* < 0.01, and *P* < 0.01, respectively).

## Discussion

Endometritis refers to a disease resulting from conditional pathogens after delivery, and *E. coli* is one of the major pathogens. This disease has caused considerable economic losses for cow producers. It has previously been reported that some probiotics can protect organisms against the damaging effects of *E. coli*, and the preliminary probiotic treatment has been demonstrated effective in the endometritis management and prevention in dairy cows (McFarland, 2014; Gärtner et al., 2015; Genís, 2016). However, the molecular mechanism beneath the antiinflammatory effects of probiotics still remains unclear (Otero et al., 2006; Yang, 2014). Hence, the *L. rhamnosus* GR-1 used in the current work was derived from the female reproductive tract to prepare an *in-vitro* model, where BEECs were subjected to *E. coli* exposure. By adopting this model, we explored the protective mechanisms whereby *L. rhamnosus* GR-1 withstands the inflammatory reactions.

The key factors for immune regulation and inflammatory response include cytokines and chemokines (Ishikawa et al., 2004; Herath et al., 2009; Ghasemi et al., 2012; Baithalu et al., 2019). Therefore, we measured the levels of proinflammatory cytokine expression, finding prominently elevated levels of IL-6, IL-8, IL-1β, and TNF-α in the *E. coli*-elicited BEECs. In addition, this study demonstrated the effectiveness of *L. rhamnosus* GR-1 in repressing the expressions of the previous proinflammatory cytokine in the *E. coli*-elicited BEECs. Confirmatively, *L. rhamnosus* exerts an antiinflammatory action against GR-1 in BEECs.

Studies have shown that *E. coli* adsorbed on BEECs through adhesion multiply in large numbers on cells and produce endotoxin, which is subsequently recognized by BEECs through TLRs (Turner, 2014). As a LPS membrane receptor, the expression of TLR4 in diverse epithelial cells is abundant (Tan et al., 2014; Chen, 2016). When the TLR4 signaling pathway is activated, signal transmission and regulation can be realized through both the MyD88-dependent and -independent pathways. Subsequently, the downstream pathways NF-κB and MAPKs are initiated (O’Neill and Bowie, 2007). Consistent with the previous studies, our study confirms that *E. coli* could increase the levels of TLR4 and MyD88 in endometrial epithelial cells. More importantly, the present study results suggested that *L. rhamnosus* GR-1 could effectively suppress the expression of these receptors’ BEECs challenged with *E. coli*. As a result, we speculate that *L. rhamnosus* GR-1 achieves modulation of *E. coli*-elicited inflammation *via* the MyD88-reliant pathway. In the future R&D and application of drugs for inflammatory disorders, the TLR4-MyD88 signaling control could be considered, and thus rewarding outcomes can be attained clinically (Wu et al., 2017).

The above-mentioned results indicate that TLR4 and MyD88 activation is probably involved in the process of endometritis inflammation. However, the precise pathway *via* which TLR4 and MyD88 regulate inflammatory responses is unclear. Several studies have demonstrated that the anti-inflammatory impacts of some probiotics depend on decreased activation of the MAPK or NF-κB signaling pathways in BEECs, and evidence has revealed that NF-κB p65, IκBα, and MAPKs-related molecules can exert the role of the target for regulating the inflammation process. For example, *Lactobacillus* GG interferes with the IκBα degradation and represses the NF-κB signal pathway initiation in Caco-2 cells (Choi et al., 2008). Lee et al. pointed out that LAB can inhibit p-IκBα, interfere with the NF-κB initiation, and represses the IL-1β and IL-6 levels in a mouse intestinal inflammation model (Lee et al., 2008). Moreover, Kim et al. found three main pathways to successfully activate the MAPKs signaling pathway in the model of colonic epithelial inflammation induced by enterotoxin (Kim et al., 2005). In line with the result of this study, the *L. rhamnosus* GR-1-repressed *E. coli* was discovered to trigger the phosphorylation of ERK1/2, JNK and p38 in BEECs, while weakening the p65 and IκBα phosphorylation. According to these approaches, NF-κB and MAPKs (JNK, ERK1/2, p38) are the mediators for the transcription of proinflammatory IL-6, IL-8, IL-1β and TNF-α cytokines in the present model system, showing consistency with previous work revealing the MAPKs and NF-κB involvement in inflammation. Together, the findings of the current work presented the suppression of proinflammatory reaction in *E. coli*-elicited BEECs by *L. rhamnosus* GR-1, which was achieved by repressing the MAPKs and NF-κB pathway initiation in these cells. Therefore, *L. rhamnosus* GR-1 holds consistent antiinflammatory functions with other probiotics.

By adopting a model system of BEECs, we demonstrated that *L. rhamnosus* GR-1 modulated the activation of upstream TLR4 and MyD88 and both downstream MAPKs and NF-κB signaling pathways, which subsequently suppressed the *E. coli*-elicited inflammatory reaction by lowering the IL-6, IL-8, IL-1β, and TNF-α levels, regulating the pro-inflammatory responses in BEECs.

## Data Availability Statement

The original contributions presented in the study are included in the article/supplementary material. Further inquiries can be directed to the corresponding author.

## Author Contributions

JL and BL performed the experiments. JL and XF drafted the manuscript. ML conceived and designed the experiments. YN critically revised the manuscript for important intellectual content. XF, YS, TJ and MF explored the data. All authors listed have made a substantial, direct, and intellectual contribution to the work and approved it for publication.

## Funding

The present project was supported by the National Natural Science Foundation of China (No. 31902328), the Specialized Research Fund for the Doctoral Program of Hebei Agricultural University (ZD201723), and the Natural Science Foundation of Hebei Province (C2021204067).

## Conflict of Interest

The authors declare that the research was conducted in the absence of any commercial or financial relationships that could be construed as a potential conflict of interest.

## Publisher’s Note

All claims expressed in this article are solely those of the authors and do not necessarily represent those of their affiliated organizations, or those of the publisher, the editors and the reviewers. Any product that may be evaluated in this article, or claim that may be made by its manufacturer, is not guaranteed or endorsed by the publisher.
